# Dopaminergic neurons differentiating from *LRRK2* G2019S induced pluripotent stem cells show early neuritic branching defects

**DOI:** 10.1038/srep33377

**Published:** 2016-09-19

**Authors:** Laurence Borgs, Elise Peyre, Philippe Alix, Kevin Hanon, Benjamin Grobarczyk, Juliette D. Godin, Audrey Purnelle, Nathalie Krusy, Pierre Maquet, Philippe Lefebvre, Vincent Seutin, Brigitte Malgrange, Laurent Nguyen

**Affiliations:** 1 GIGA-Research, GIGA-Neurosciences, Université de Liège, Belgium; 2 Service de Neurologie, CHU Sart Tilman, Belgium; 3 Service d’othorhinolaryngologie, CHU Sart Tilman, Belgium

## Abstract

Some mutations of the *LRRK2* gene underlie autosomal dominant form of Parkinson’s disease (PD). The G2019S is a common mutation that accounts for about 2% of PD cases. To understand the pathophysiology of this mutation and its possible developmental implications, we developed an *in vitro* assay to model PD with human induced pluripotent stem cells (hiPSCs) reprogrammed from skin fibroblasts of PD patients suffering from the *LRKK2* G2019S mutation. We differentiated the hiPSCs into neural stem cells (NSCs) and further into dopaminergic neurons. Here we show that NSCs bearing the mutation tend to differentiate less efficiently into dopaminergic neurons and that the latter exhibit significant branching defects as compared to their controls.

Parkinson’s disease (PD) is a neurodegenerative disorder affecting more than 1% of the global population over the age of 70^1^. PD is mainly characterized by the progressive loss of dopaminergic (DA) neurons of the ventral midbrain in the *substantia nigra pars-compacta* (SNpc) that project to the striatum^2^,^3^. This neuronal loss leads to an overall decrease of dopamine release in the striatum and to motor dysfunctions such as tremor, rigidity and akinesia^4^. Most PD patients suffer from sporadic forms of the disease, thus familial forms account for only about 10% of all cases^5^,^6^. Several genes and multiple *loci* have been associated with the heritable forms^7^,^8^ and in particular, the mutation of the *leucine rich repeat kinase 2 (LRRK2*) gene, which is the most commonly mutated gene associated with dominant familial PD^9^,^10^,^11^. Six pathogenic mutations have been identified in *LRRK2*^12^,^13^, one occurring at a high rate in hereditary PD: the Gly2019Ser (G2019S)^10^,^14^. *LRRK2* encodes a large protein containing a central catalytic domain, combining a GTPase domain called ROC domain (Ras of complex domain), and a kinase domain. It also bears a N-terminal leucine rich repeat region and a C-terminal WD40 domain that mediates protein-protein interaction. The dominant mutations of *LRRK2* associated with PD are found in the core catalytic domain - for example, the G2019S mutation is located at the level of the kinase domain. LRRK2 auto-phosphorylates at the level of its ROC domain to regulate its own activation^15^,^16^ and the mutation results in an increased kinase activity^17^,^18^,^19^. In addition to its protein-protein interactions and putative scaffolding role, LRRK2 interacts with and phosphorylates microtubules (MT) *in vitro*^20^. It was shown that MT phosphorylation promotes their stabilization and this activity is increased 3-folds by the G2019S mutant protein^21^. This LRRK2-related activity suggested that it may contribute to neuritogenesis, which was further supported by the observation that loss of LRRK2 expression increases neurite length both *in vivo* in mice and *in vitro*^22^. Moreover, the *LRRK2* G2019S mutation results in the shortening of neurites^21^,^23^,^24^. The toxicity of the *LRRK2* G2019S mutation towards DA neurons of PD *patients* may arise from deficient autophagy, in some cases evidenced by alpha-synuclein accumulation^25^ or from increased oxidative stress^26^,^27^. Given the broad expression of LRRK2 in various types of neurons, it is currently unclear why the *LRRK2* G2019S mutation impairs more specifically the viability of DA neurons.

To model PD, human induced pluripotent stem cells (hiPSC) have previously been derived from skin biopsies of patients bearing the *LRRK2* G2019S mutation or their controls^28^,^29^. In these studies, DA neurons were differentiated from the hiPSCs and showed PD related phenotypes such as the presence of autophagic vacuoles and reduced neurite number and length after long-term culture (75 days)^25^.

To test whether early developmental defects could be observed in hiPSCs-derived DA neurons bearing the *LRRK2* G2019S mutation, we reprogrammed hiPSCs from three healthy donors and three unrelated patients diagnosed with familial PD carrying the G2019S mutation in the *LRRK2* gene. Neural stem cells (NSCs) derived from these hiPSCs were further differentiated into DA neurons according to a newly developed feeder-free fast protocol. Using this novel approach, we were able to show that at early stages of differentiation, the *LRRK2* G2019S differentiating DA neurons harbor a significant reduction of total neuritic length and a more complex neuritic tree as compared to controls. Our results suggest the existence of early morphological defects in DA neurons in *LRRK2* G2019S mutated patients.

As the axonal arborization size could play a major part in the selective vulnerability of DA neurons^30^, this mutation may increase the sensitivity of mature DA neurons to toxic events leading to the death of DA neurons during adulthood in PD patients.

## Materials and Methods

### Human ES culture

Human ES cell research and protocols were approved by the Ethics Committee of the University of Liège (#B70720096466). All experiments were conducted according to its guidelines. Human ES H9 cells (WA-09, WiCell Research Institues, Madison USA, MTA agreement number 10-W0146) were maintained on gamma ray-irradiated mouse embryonic fibroblasts (MEFs) cultured in DMEM/F12 medium supplemented with 20% of knockout serum replacement (KSR, Invitrogen), 100 μM non-essential amino acids, 100 μM 2-mercaptoethanol and 4 ng/ml basic Fibroblast Growth Factor (bFGF) (Peprotech, London U.K.). The cells were mechanically passaged weekly.

### hiPSC derivation

Approvals of the Ethics Committee of the University of Liège for research and protocols (#B70720096309), and patient informed consents were obtained before deriving hiPSCs from skin fibroblasts isolated by punch biopsies. All experiments were conducted according to the guidelines of the Ethics Committee of the University of Liège. Three biopsies came from healthy donors (WT) and two from PD patients with confirmed *LRRK2* G2019S mutation. One PD (G2019S) fibroblast cell line was purchased (ND29370, Coriell Institute). Dermal fibroblasts were amplified by three passages and then reprogrammed using Sendai virus vectors (Cytotune iPS reprogramming kit, Life Technologies Invitrogen). The pluripotent state of the colonies was validated by immunohistochemistry and qRT-PCR to detect expression of endogenous Nanog, Oct4, Sox2 and Tra-1-81. Moreover, the pluripotency of the reprogrammed cells was assessed *in vivo* by subcutaneous flank injection of Nod/Scid mice (Jackson Laboratory) to generate teratoma-encapsulated tumours. Tissues from the three germ layers: mesoderm (primitive cartilage, muscles, fat), endoderm (primitive gut like epithelium) and ectoderm (immature squamous epithelium, neural rosettes) were identified after haematoxylin and eosin coloration.

### Induction of human neural stem cells

The H9 and hiPSCs colonies were enzymatically detached from MEFs with Collagenase A treatment at 1 mg/ml (Roche, Belgium) for 20 minutes and plated 6 hours in non-adherent conditions in DMEM/F12, 2% of B27 without vitamin A (12587-010 Gibco) and supplemented with: 1% of N-2 Supplement (17502-048 Gibco), 10 μM of Y-27632 (ROCK inhibitor; Tocris Biochem), 500 ng/ml of noggin (120-10C Peprotech), 20 μM of SB431542 (SMAD inhibitor; S4317-5MG Sigma) and bFGF 2 ng/ml. Cells were plated 10 days on Poly-ornitin/laminin (P4638-1G Sigma; L2020-1MG Sigma) coated dishes in this medium before being detached with trypsin and re-plated on Poly-ornitin/laminin coated dishes and cultured in the neural induction medium: 50–50% DMEM/F12 - Neurobasal medium supplemented with 2% of B27, 1% of N-2, 0.5% Glutamax (35050-038 Gibco), 10 ng/ml of Epidermal Growth Factor (EGF; AF-100-15 Peprotech) and bFGF 10 ng/ml. Culture of cells in this neural induction medium generates homogenous cultures of NSCs (more than 95% of the cells).

### DA neuron progenitor derivation

NSCs were grown at high confluency (70%) for 7 days on Poly-ornitin/laminin coated dish in DMEM/F12 with 1% of N2 supplement, 200 ng/ml of Sonic Hedgehog; 100 ng/ml of Fibrobalst Growth Factor 8 (FGF8). This first culture step was required to convert NSCs into DA neurons progenitors.

### DA neuron differentiation

DA progenitors were plated on Poly-ornitin/laminin coated dish, in DMEM/F12, 1% of N2 supplement, 20 ng/ml of Brain Derived Neurotrophic Factor (BDNF; 450-02, Peprotech), 0.2 mM of ascorbic acid (AA; A5960-25G, Sigma), 20 ng/ml of GDNF (450-10, Peprotech), 0.5 mM of dibutyryl cbcAdenosine Mono Phosphate (dbcAMP; D0627-25MG, Sigma), 1 ng/ml of Tranforming Growth Factor beta3 (TGFb3; 100-36E, Peprotech). At day 7, the medium was swapped by Neurobasal medium (21103-049, Gibco) supplemented with 2% of B27, 1% of N2 and BDNF, AA, GDNF, dbcAMP, TGFb3 (concentrations, same as above). The differentiating DA neurons were further kept up to 35 days in the same medium which was renewed every week.

### Whole-cell electrophysiological recordings

During recordings, cover slips were continuously superfused with artificial cerebrospinal fluid (ACSF; 140 mM NaCl, 5 mM KCl, 2 mM CaCl_2_, 2 mM MgCl_2_, 15 mM HEPES and 10 mM D-glucose; pH 7.4) heated at 32 °C using a Thermoclamp (Automate scientific, Berkeley, USA). ACSF and drug applications were performed using gravity and a BPS-8 valve control system (ALA Scientific, Westbury, NY, USA). 0.5 μM tetrodotoxin (TTX; Tocris Biosciences, Ellisville, MO, USA), 20 mM tetraethylammonium (TEA; Sigma-Aldrich) and 30 μM ZD7288 (Tocris Biosciences, Ellisville, MO, USA) were added to the ACSF in some experiments to block Na^+^, K^+^ or I_h_ currents respectively; 1 μM quinpirole hypochloride (Tocris Biosciences, Ellisville, MO, USA) was applied to check for the presence and functionality of D2 autoreceptors (the activation of which hyperpolarizes canonical DA neurons by opening G-protein coupled inwardly rectifying K^+^ channels). Pipettes were pulled on a P-87 micropipette puller (Sutter Instruments, Novato, CA, USA) using borosilicate glass capillary tubing (2.0 mm OD 1.16 mm ID; Hilgenberg, Malsfeld, Germany). The resistance of the electrodes was 5–8 MΩ when filled with the following solution: 130 mM KGluconate, 10 mM KCl, 0.5 mM CaCl_2_, 15 mM HEPES, 8 mM NaCl, 2 mM ATP-Mg, 0.3 mM ATP-Na_2_, 5 mM EGTA and 11.1 mM D-glucose; pH 7.4.

Neurons were visualized using an Axiovert microscope (Zeiss, Oberkochen, Germany). When a gigaohm seal was obtained, application of negative pressure was applied to obtain the whole-cell configuration. Membrane potentials and currents were recorded using an EPC9 amplifier (HEKA, Lambrecht/Pfalz, Germany) connected to Patchmaster software (HEKA, Lambrecht/Pfalz, Germany). Liquid junction potentials were corrected. Only recordings in which the series resistance was lower than 30 MΩ and remained stable for the duration of the recording (variations ≤ 20%) were used. No compensation of the series resistance was performed.

### Quantitative RT- PCR analyses

Total RNA was obtained from culture dishes using RiboPure™ RNA Purification Kit (Life Technologies) and following the manufacturer’s instructions. RNA quantity and quality was assessed using the NanoDrop 1000 (Nano- Drop Technologies). cDNA was synthesized using Superscript III first strand synthesis kit (Invitrogen). Quantitative PCR was performed on a LightCycler 480 (Roche) using SYBR GreenER Super-MIX (Invitrogen). Annealing temperature was optimized for each primer set and the PCR reactions were evaluated by melting curve analysis. Human GAPDH and PPIA mRNA were amplified to ensure cDNA integrity and to normalize expression. Quantitative PCR array for human autophagy was performed using RT^2^ Profiler PCR Array (#PAHS-084ZA; Quiagen); n = 1.

### Immunocytochemistry

Cells were fixed in 4% of paraformaldehyde in Phosphate Buffer Saline (PBS), blocked for 30 min in PBS supplemented with 5% of normal donkey serum and 0.3% of triton X-100. Primary antibodies used were directed against FoxA2 (sc-6554, 1/100, Santa Cruz,); Ki67 (550609, 1/250, BD); Lmx1a (AB10533, 1/250, Millipore); Map2 (M2320, 1/500, Sigma); Nanog (AF1997, 1/250, R&F), Nestin (NB100-1604, 1/500, Novus Biologicals); Oct4 (sc-5279, 1/500, Santa Cruz); Pax6 (AB-528427, 1/1000, DSHB); Sox1 (AF3369, 1/500, R&D); TH (CH23006, 1/500, Acris Antibodies GmbH); Tra-1-81 (MAB4381, 1/500, Millipore); or βIII tub (MMS-435P, 1/1500, Covance). Nuclei were counterstained with Dapi (1/1500). Immunocytochemistry preparations were imaged using a Nikon A1 confocal microscope.

### Sholl analysis

DA progenitors were nucleofected with an eGFP encoding plasmid at 2 μg/μl using the Amaxa neuronal kit (Lonza, VPG-1001). The cells were then cultured under DA neuron differentiation conditions and were then fixed with 4% PFA after 1, 3 or 5 days of differentiation. GFP positive neurons were imaged using a Nikon A1 confocal microscope. Images of individual cells were manually cleaned from surrounding background and the arborization complexity was measured by counting the number of neurite intersections with concentric circles radiating from the cell body. This was measured with the Sholl plugin in ImageJ software (Bethesda, MD, USA).

### Microtubule polymerization assay: comet assay

DA progenitors were nucleofected with pCMV-EB3-GFP plasmids (2 μg/μl). The cells were then cultured by following the DA neuron differentiation protocol. Cells were imaged every 1 s for a total duration of 1 min using the resonant imaging mode on a Nikon A1 confocal. The MT polymerization speed was measured using the Multiple Kymograph plugin on ImageJ. Comet movement was measured from the tip of each comet and velocity was quantified as length traveled (μm) versus time (s)^31^.

### Statistics and counting

For quantification of immunolabelled cells, countings were performed in randomly chosen imaged fields (average of 20 fields counted per experiment). Data points represent the average of at least three independent experiments. For statistical analysis of cell numbers and qPCR at different culture time points, one-way Anova with Bonferroni’s multiple comparison post-test was used to determine the significance of the results (using GraphPad Prism, version 5.0d). Total neurite length was compared using a Student’s t-test. For Sholl analysis, independent samples t-tests were used to compare groups of distances (0 to 100 μm and 100 to 200 μm day 1; 0 to 200 μm and 200 to 400 μm day 3; 0 to 300 μm and 300 to 700 μm day 5) in WT vs mutant. Unless noted otherwise, all data represent the mean ± SEM.

## Results

### Protocol for fast and efficient derivation of ventral midbrain dopaminergic neurons from human pluripotent stem cells

**Figure 1:**
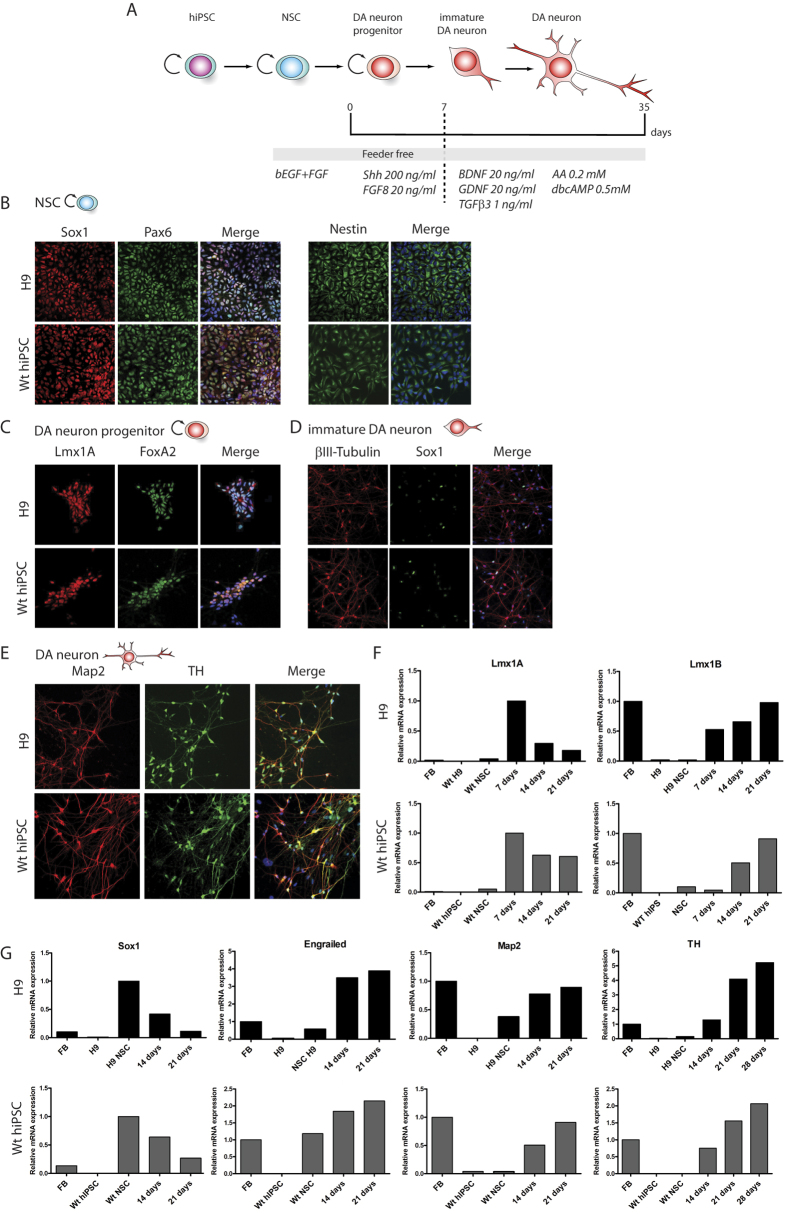
Dopaminergic neuron derivation from H9 stem cells and hIPSCs. Scheme showing the different steps of the DA neuron derivation protocol (**A**). Immunolabelings of cultured H9 (top panels) and WT hiPSCs (bottom panels) for different NSCs markers, Sox1, Pax6 and Nestin (**B**). Immunolabeling of cultured H9 (top panels) and WT hiPSCs (bottom panels) after 3 days of DA progenitor induction for progenitor markers Lmx1A and FoxA2 (**C**) and after 7 days of induction for immature neurons βIII-Tubulin+ and remaining progenitors Sox1+ (**D**). Immunolabelings of cultured H9 (top panels) and WT hiPSCs (bottom panels) after 21 days of induction for the neuronal marqueur MAP2 and DA neurons Tyrosine Hydroxylase (TH) (**E**). Analysis of gene expression levels by qRT-PCR for cultured H9 (top panels) and WT hiPSCs at different time points (NSC, 14 days and 21 days) of the culture (FB = fetal brain) (**F**,**G**).

We developed a novel *in vitro* protocol for the rapid and efficient generation of DA neurons from human ES or hiPSCs, collectively named “pluripotent stem cells”. The protocol was first established using H9 cells and was further validated with different hiPSC lines. The first step of differentiation toward the dopaminergic fate was the generation of NSCs (Fig. 1A). For this purpose, we cultured pluripotent stem cells for 10 days in the neural induction medium (see material and methods). We performed immunolabelings as well as qRT-PCRs to check the phenotype of the newly generated NSCs. Cells derived from both H9 and hiPSCs homogenously expressed a typical combination of neuroectodermal markers including the transcription factor Sox1, the proliferation marker Ki67 (not shown) and the intermediate filament protein Nestin (Fig. 1B,G). The NSCs could be amplified as monolayers and passaged up to 10 times while retaining their neuronal progenitor cells (NPCs) characteristics (data not shown). The NSCs represent an important step in the differentiation protocol as these cells can be amplified and frozen, thus allowing starting all DA neuron differentiation protocols from the same pool of NSCs. The previously established DA neuron differentiation protocols usually require the formation of embryoid bodies in floating culture conditions for 4 to 7 days followed by placing the cells in adherent condition to for neuronal rosettes^32^,^33^.

The next step was to generate DA neurons that show ventral midbrain characteristics by differentiation of NPCs derived either from H9 or hiPSCs. NPCs were cultured for 7 days with a DA neuron progenitor induction medium (Fig. 1A and material and methods). The resulting cells expressed the ventral midbrain determinants Lmx1A/B and FoxA2, transcription factors expressed in DA neuron progenitors^34^,^35^,^36^ (Fig. 1C).

DA neuron progenitors were further cultured in the DA neuron differentiation medium (see material and methods). Immunolabelings supported the induction of neuronal differentiation as shown by the increase of cells expressing the neuron-specific class III-β-Tubulin (βIII-Tubulin) at the expense of cells expressing the neuroectodermal marker Sox1 (Fig. 1D). The reduction of Sox1 expression was confirmed by qRT-PCR analyses (Fig. 1G). The progressive differentiation of these neurons into ventral midbrain DA neurons was further supported by a progressive reduction of Lmx1A expression concomitant with the increased expression of a combination of DA markers including Engrailed and tyrosine hydroxylase (TH) together with βIII-Tubulin and Map2 (Fig. 1E–G).

We next characterized the functional maturation of NSCs into DA neurons by performing electrophysiological recordings at different maturation time points. At 10 days of culture DA neuron progenitors fired either spontaneously (Figure S1A) or following current injection (Figure S1B). In addition, typical fast Na^+^ (blocked by TTX) and slower K^+^ currents (blocked by TEA) were evoked in voltage clamp recordings in both types of cultures (Figure S1C). After 20 days of culture, two characteristic features of mature DA neurons appeared in cultures issued from both hES and hiPSCs. First, application of quinpirole, a D2 agonist, gave rise to a reversible hyperpolarization (Figure S1D) indicating the presence of functional D2 autoreceptors. Second, hyperpolarizing current injections produced a “sag” in the voltage deflexion, a characteristic feature of mature DA neurons resulting from the presence of the cation mixed I_h_ current. As expected, this sag was blocked by addition of the I_h_ blocker ZD7288 (Figure S1E)^37^.

From these experiments, we conclude that our protocol allows the fast (between 27 and 42 days *in vitro*) and efficient generation of neurons from either H9 or hiPSCs that show typical immunogenic and electrophysiological phenotypes of midbrain-derived DA neurons that can be maintained for up to 35 days. The yield of about 20 to 30% DA neurons of our protocol is higher or comparable to the previously published iPSCs differentiation protocols but it is faster as it bypasses the step of embryoid bodies and rosettes formation (for a reviewed comparison of DA neuron differentiation protocols^38^).

### Generation of hiPSCs from monogenic forms of PD

Skin punch biopsies were performed on PD patients diagnosed with the *LRRK2* G2019S mutation and on control subjects with no known PD-related mutations or any other neurological disorder; additional mutant fibroblasts were purchased from Coriell (ND29370; Coriell Institute). Dermal fibroblasts grown out of biopsy were reprogrammed at passage 3 into hiPSCs. Several hiPSCs lines were generated for each individual. Overall, 3 controls and 3 *LRRK2* G2019S cell lines were fully characterized and used for DA neuron differentiation (Figure S2A).

Mutation of *LRRK2* at G2019 did not interfere with the reprogramming of fibroblasts, and the presence of the mutation was verified by DNA sequencing (Figure S2B). All hiPSC lines exhibited similar morphology to H9 and could be maintained in proliferation for long-term passaging (>50) onto irradiated mouse embryonic fibroblasts (MEFs) feeder layer. After reprogramming, the hiPSCs were characterized by the expression of endogenous pluripotency markers (Oct4, Sox2, Nanog and TRA-1-81) as reported by immunolabelings and qRT-PCR (Figure S2C,D). In addition, karyotyping supported chromosomal integrity in all cell lines (Figure S2E and data not shown). Thus, the newly generated hiPSCs were *bona fide* pluripotent stem cells, and no differences were observed between healthy and *LRRK2* G2019S PD cell lines in their reprogramming efficiency or maintenance.

### WT and PD patient-derived hiPSCs generate DA neurons in culture

**Figure 2:**
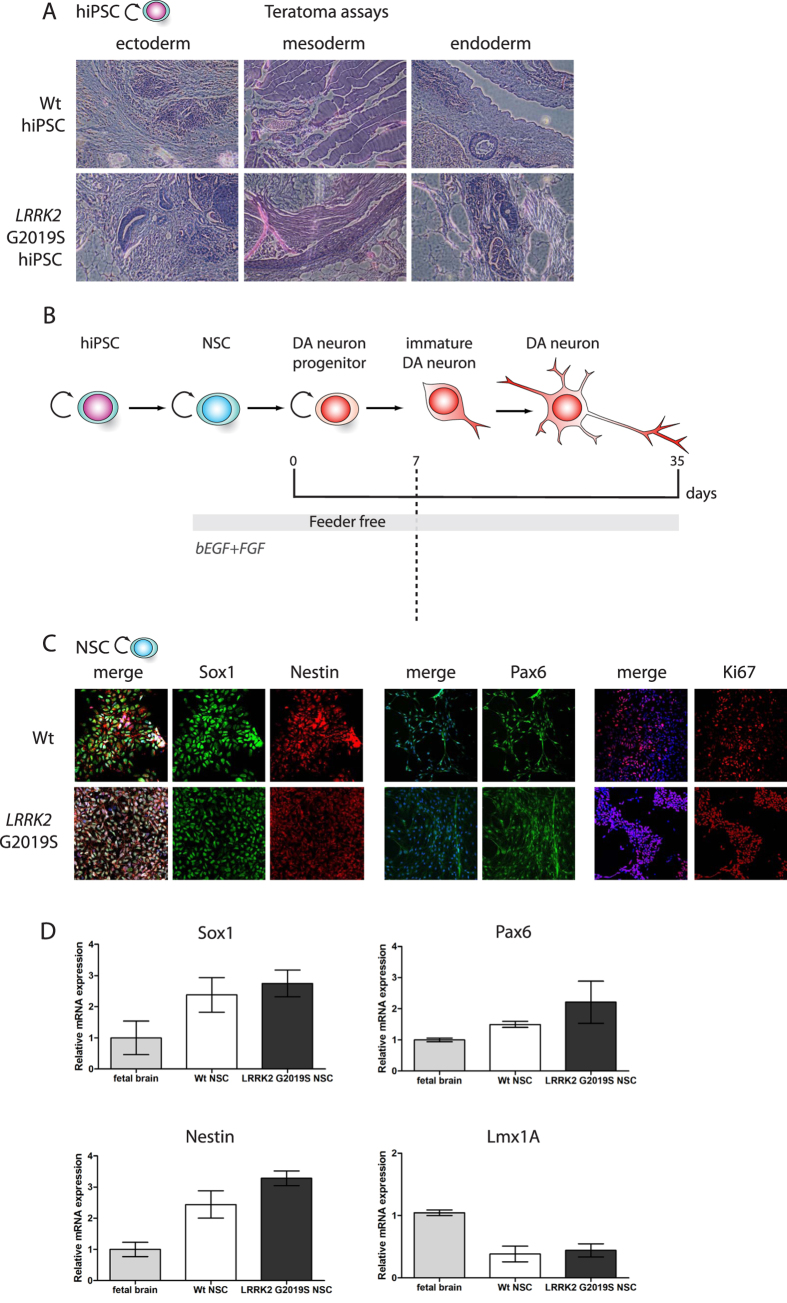
Neural Stem Cell derivation from WT and LRRK2 G2019S hiPSCs. Haematoxylin and eosin coloration of teratoma-encapsulated tumors generated by flank injection of WT and G2019S hiPSCs cells in Nod/Scid mice. Representative images of the different germ layers: ectoderm (immature squamous epithelium, neural rosettes), mesoderm (primitive cartilage, muscles, fat) and endoderm (primitive gut like epithelium) (**A**). Scheme showing the different steps of the DA neuron derivation protocol (**B**). Immunolabelings of cultured WT and *LRRK2* G2019S hIPSCs for different NSCs markers after NSCs induction protocol: Sox1, Nestin, Pax6 and Ki67 (**C**). Analysis of gene expression levels by qRT-PCR for WT and *LRRK2* G2019S cultures at the end of the NSCs induction protocol (**D**).

**Figure 3:**
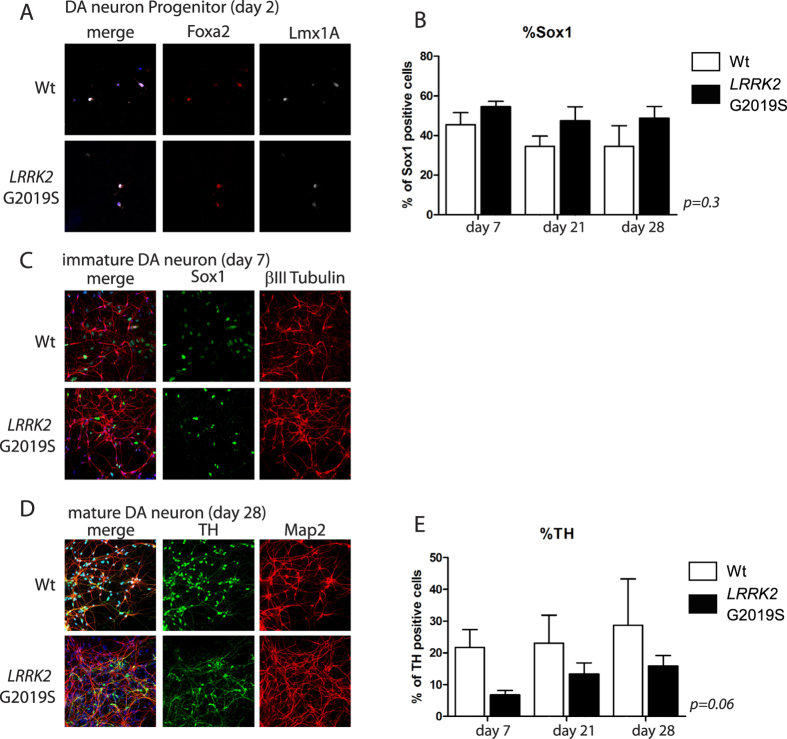
Dopaminergic neuron differentiation from Wt and LRRK2 G2019S NSCs . Immunolabeling of cultured WT (top panels) and *LRRK2* G2019S (bottom panels) cells after 3 days of DA progenitor induction for progenitor markers Lmx1A and FoxA2 (**A**). Histogram of the percentage of Sox1+ cells amongst Dapi stained nuclei at different time points (day 7, 21, 28) of the culture (**B**). Immunolabeling of cultured WT (top panels) and *LRRK2* G2019S (bottom panels) cells in differentiation condition for marker of neuronal differentiation (βIII-Tubulin) or neuroectodermal progenitor (Sox1) after 7 days of induction (**C**). Immunolabeling of cultured WT and *LRRK2* G2019S cells at the end of the differentiation protocol (day 28) for markers of neurons (MAP2) and DA neurons (Tyrosine Hydroxylase, TH) (**D**). Histogram of the percentage of TH+ cells at different time points (day 7, 21, 28) of the culture amongst the MAP2+ cells (**E**).

All six hiPSC lines were able to generate teratoma-encapsulated tumors in immunodeficient mice confirming their pluripotency (Fig. 2A). Using our derivation protocol, hiPSC lines generated were differentiated into NSCs (Fig. 2B). The NSCs lines were propagated as monolayer cultures for many passages with no differences between WT and *LRKK2* G2019S regarding their proliferation potential (data not shown). Moreover, the efficiency of differentiation into NSCs was comparable between WT and *LRKK2* G2019S hiPSC. Immunostainings and qRT-PCRs were performed to confirm the silencing of the reprogramming transgenes (data not shown). The NSC identity of the differentiated cells was validated by immunolabelings and qRT-PCR. These cells harbored a combination of neuroectodermal markers (Sox1, Pax6 and Nestin) whose expression was comparable to the one measured in human fetal brain (Fig. 2B,C). Expression of the DA progenitor marker Lmx1A (as assessed by qRT-PCR) was weak as compared with fetal brain (Fig. 2C) and not observed by immunostainings (data not shown), supporting no spontaneous DA neuron commitment in the NSCs cultures. We next cultured NSCs in the DA neuron induction medium and monitored their commitment and differentiation into midbrain-derived DA neurons at different timings of culture (day 7, 21 and 28). We first assessed the percentage of the residual progenitors (Sox1+) as the cultures matured (Fig. 3B) by immunostaining (Fig. 3C for day 7). While we observed a trend of progressive reduction of Sox1 expression in differentiating WT NSCs, there was a tendency for Sox1+ cells to persist in LRRK2 G2019S cultures (Fig. 3B,C). We then assessed the expression of TH as marker of dopaminergic differentiation and tested the ability of WT and LRRK2-G2019 NSC cultures to give rise to DA neurons. NPCs progressively gave birth to neurons expressing Map2 amongst which neurons were TH+. Again, we observed a trend of WT NPCs to give birth to more TH+ neurons as compared to their LRRK2-G2019 counterparts (Fig. 3D,E). The general morphology of these TH+ cells was homogeneous in the cultures; they presented a long neurite that progressively arborized over the course of the culture (Fig. 3D,E). Although not significant, the reduced amount of TH+ cells in culture was in agreement with the higher number of persisting progenitor cells in the *LRRK2* G2019S. Nonetheless, both types of cell lines were able to generate a high percentage of DA neurons in culture allowing us to study their early differentiation steps.

### Early differentiating LRRK2 G2019S mutant DA neurons show a neuritic hyperbranched phenotype

As PD is a late onset neurodegenerative disease, several groups have characterized cell cultures of DA neurons after long-term culture to look for naturally occurring signs of neurodegeneration such as: formation of cellular aggregates of alpha-synuclein, number and length of neurites, and level of oxidative stress^25^,^30^,^39^,^40^.

**Figure 4:**
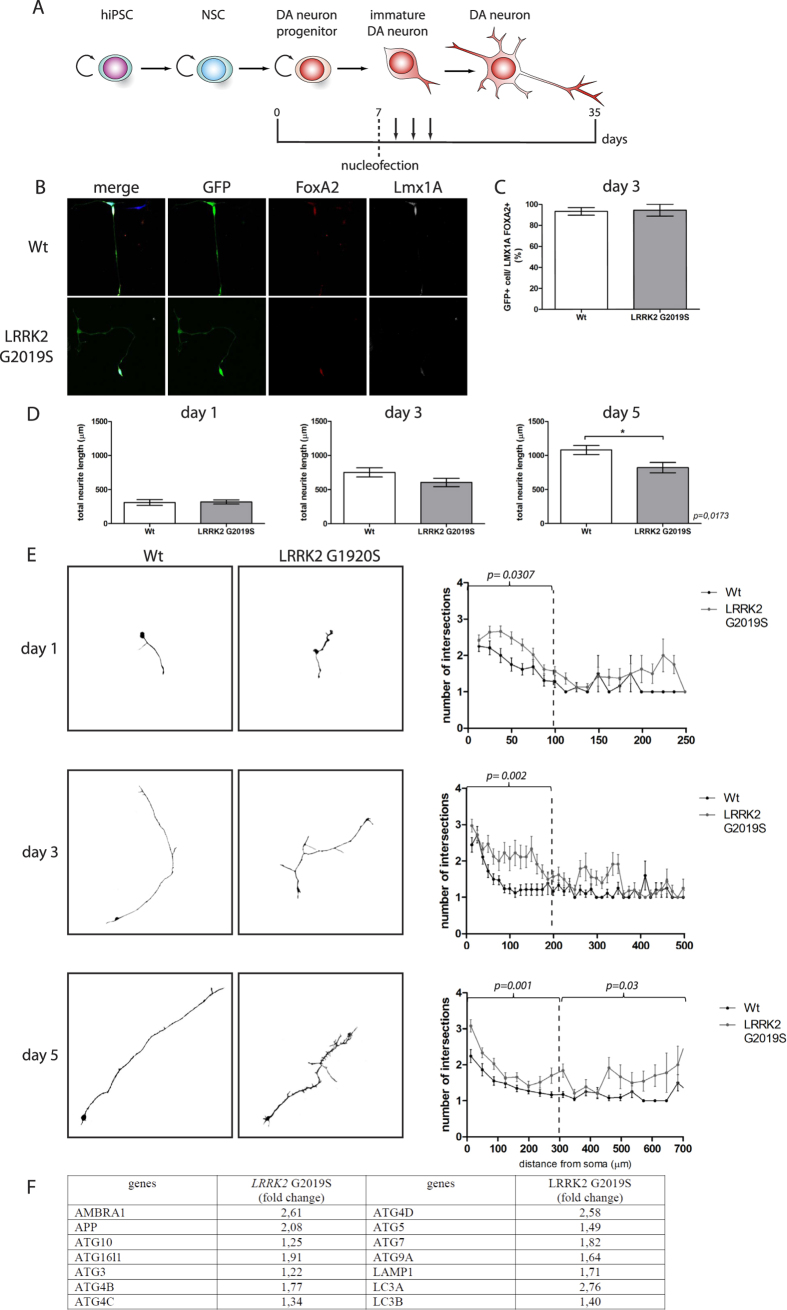
LRRK2 G2019S derived dopaminergic neurons show defects of neuritic length and complexity. Scheme showing the different steps of DA neuron derivation protocol. Black arrows represent the different measured time points after nucleofection (day 1, 3 and 5) (**A**). Immunolabeling of nucleofected WT and *LRRK2* G2019S differentiating cells for marker of DA progenitor: FoxA2 and Lmx1A and for nucleofection marker GFP (**B**). Histogram of the percentage of GFP cells co-labelled with FoxA2 and Lmx1A at 3 days post nucleofection of the WT and *LRRK2* G2019S cultures (**C**). Sum of the length of all neurites in a cell, called total neuritic length of WT and *LRRK2* G2019S differentiating DA neuron in μm, at different time points of the culture after nucleofection (**D**). Sholl processed images, representative of the morphology of WT and *LRRK2* G2019S differentiating DA neurons (left panels). Sholl quantifiquation of neuronal complexity at different time points of the culture for WT and *LRRK2* G2019S differentiating DA neurons (right panel). All 3 WT and 3 mutant cell lines were pulled for the analysis. Day 1 n = 24 for WT and n = 50 for mutant; Day 3 n = 18 for WT and n = 24 for mutant; Day 5 n = 29 for WT and n = 36 for mutant (**E**). qRT-PCR array summary of autophagy genes differentially expressed between WT and *LRRK2* G2019S cell cultures at 7 days of differentiation. The fold change values in the table represent the variation of the *LRRK2* G2019S condition compared to the WT condition normalized to 1.

Although, short-term cultures of DA neurons were not reported to have branching defects, no detailed morphological analyses were performed^25^,^29^. In order to test if early developmental defects could be seen in genetically encoded PD cell lines, we characterized the morphological properties of healthy and PD patient DA neurons in culture at early time points of differentiation. For this purpose, we nucleofected DA neuron progenitors with GFP expression plasmids and monitored the first steps of differentiation (1, 3 and 5 days after transfection) (Fig. 4A). As a control, 3-day-old cultures were immunolabeled for early markers of DA progenitors, such as FoxA1 and Lmx1A, to ensure the proper commitment of the NSC. No differences were observed between WT and *LRKK2* G2019S cell lines. Most nucleofected cells were positive for both FoxA1 and Lmx1A (Fig. 4B,C) (93,27% ± 3 for WT cultures and 94,33% ± 5 for PD cultures). Of note, at day 5 the number of cells expressing TH was very low, so the DA identity of the differentiating GFP cells could only be inferred by the expression DA progenitor markers at earlier time points of the culture. We assessed the early steps of neurite branching, focusing on the total neurite length (sum of all neurites length in a cell), a typical hallmark described for *LRRK2* G2019S mutant DA neurons in aged cell culture^22^,^23^,^25^. We observed a significant reduction of total neuritic length for *LRRK2* G2019S DA neuron differentiating NSCs (expressing Lmx1A) (821,60 μm ± 75,10) as compared with their controls (1080 μm ± 66.89) 5 days post nucleofection (P = 0.0173), but not earlier (Fig. 4D).

Differentiating DA neurons were then assessed for their neuritic tree complexity by Sholl analyses performed at 1, 3, and 5 days after GFP nucleofection (Fig. 4E). Our data showed that the differentiating cells derived from the *LRRK2* G2019S NSCs were more complex at all the time points analyzed (at day1: 1.26 ± 0.07 for WT culture and 1.70 ± 0.11 for PD culture, p = 0.0307; at day 3: 1.53 ± 0.12 for WT culture and 2.2 ± 0.09 for PD culture in the proximal regions, p = 0.002; at day 5: 1.11 ± 0.03 for WT culture and 1.77 ± 0.12 for PD culture at proximal and distant regions from the soma p = 0.001 and p = 0.03, respectively (Fig. 4E)). This phenotype has not been described before and is not concordant with the neurodegeneration phenotype described for *LRRK2* G2019S mutant DA neurons, which harbor a reduced neurite length and complexity at later stages of long-term cultures^25^,^39^.

LRRK2 is a kinase that plays a major role in cytoskeletal dynamics through the association and regulation of Tau activity, promoting MT assembly^41^,^42^. In order to check whether the early neuritic defects seen upon *LRRK2* G2019S mutation involve the impairment of the microtubule cytoskeleton, we nucleofected differentiating DA neuron progenitors with end-binding protein 3 (EB3)-GFP expressing constructs. EB3 is a plus tip microtubule-associated protein that labels the fast growing end of microtubules^43^. Time-lapse movies were acquired at different time points of differentiation (1, 2 and 3 days after nucleofection) and the microtubule polymerization speed was measured. At all the different culture time points measured, the MT polymerization rate was similar in WT and LRRK2 G2019S cultures (day1: 0.225 ± 0.006 μm/s for WT cells, 0.227 ± 0.008 μm/s for mutant; day 2: 0.215 ± 0.006 μm/s for WT, 0.207 ± 0.009 μm/s for mutant; day 3: 0.208 ± 0.006 μm/s for WT, 0.204 ± 0.008 μm/s for mutant cultures) (Figure S3A,B).

Autophagy induction is implicated in toxin-induced PD^44^ and contributes to shortening of neurites in *LRRK2* G2019S cell line^23^. Therefore, we tested its implication at early stages of our differentiation model by performing a human autophagy quantitative RT-PCR array at 7 days of differentiationbetween WT and *LRRK2* G2019S (n = 1). Interestingly several marquers of autophagy were upregulated such as microtubule-associated protein Light Chain 3(LC3A), ATG7, and ATG5 present on autophagic vesicles as well as LAMP1 associated with lysosomes (Fig. 4F). These preliminary results indicate that autophagy induction in the *LRRK2* G2019S mutated cell line could be the cause of the neuritic phenotype observed during DA neuron differentiation.

## Discussion

In this study, we describe a novel differentiation protocol for the fast and efficient differentiation of pluripotent stem cells into midbrain DA neurons (over 20% of TH+ DA neurons in a homogenous βIII-tubulin+ neuronal population). These neurons exhibit classical immunogenic and physiological phenotypes of midbrain DA neurons. By using this protocol, we have generated and characterized several novel hiPSCs cell lines from PD patients bearing the *LRRK2* G2019S mutation as well as from healthy controls. We have demonstrated that DA neurons differentiated from *LRRK2* G2019S PD patient-derived hiPSCs have the same ability to differentiate into DA neurons, although they display morphological differences at early stages of differentiation as compared to their controls. After 1, 3 and 5 days of neuronal differentiation, the *LRRK2* G2019S cells display a more complex neuritic arborization as well as shorter total neurite length at day 5 as compared to their controls. This is similar with the described phenotype at older time points of the cell culture, in which reduced neuritic length is a hallmark of *LRRK2* mutations in DA neurons^21^,^22^,^23^. It has been proposed that LRRK2 controls actin cytoskeleton dynamics through putative effectors such as ezrin, radixin, and moesin (ERM) and that increased LRRK2 kinase activity, resulting from the G2019S mutation, was correlated with more phosphorylation of the target ERM proteins^24^. LRRK2 has also been shown to regulate the association of Tau protein to the microtubules where it promotes MT assembly^41^,^42^. It is interesting to note that the defect in elongation and complexity of neurites is not dependent on the MT polymerization rate - as suggested by the EB3 comet speed measurement using live cell imaging. It is noteworthy that at 1, 3 and 5 days of culture no aggregation of neurite were observed, in contrast to what is seen in sensory neuron cultures^45^. Autophagy has been implicated in the maintenance of neurite length and defective autophagy could be implicated in the neuropathology of PD^25^,^46^,^47^. In accordance with this hypothesis, we detected by qRT-PCR induction of autophagy in our mutant cell line at 7 days of differentiation. This induction could occur early on during the differentiation process, giving rise to neuritic length defects in the *LRRK2* G2019S DA neurons. The observation that mutant neurons showed increased branching complexity at early developmental stages is novel and in striking contrast with what has been reported in aged PD DA neurons showing signs of degeneration including reduced neurite number and complexity^25^,^39^. The decreased branching complexity observed at later stages in DA neurons differentiated from PD hiPSCs is thought to arise from a cellular pathotoxicity resulting from increased oxidative stress and impaired autophagy^25^. Recently, it has been hypothesized that the axonal arborization size plays a major part in the selective vulnerability of DA neurons^48^,^49^,^50^. DA neurons from the SNc display a very dense and complex axonal arborization as compared with ventral tegmental area DA neurons, and these morphological characteristics are accompanied by elevated basal oxidative phosophorylation and ROS production due to the high demand of ATP^30^,^51^. The increase in neuritic arborization complexity of *LRRK2* G2019S DA neurons that we describe in this work may correspond to an early developmental defect that increases the cellular vulnerability, through elevated ATP demand and ROS production, which may contribute later to the specific degeneration of DA neurons.

Our novel and fast differentiation protocol offers an excellent tool to understand the physiopathology underlying LRRK2 mutations and could help us understand the molecular links between cytoskeleton regulation, neuronal morphology and neurodegeneration.

## Additional Information

**How to cite this article**: Borgs, L. *et al.* Dopaminergic neurons differentiating from *LRRK2* G2019S induced pluripotent stem cells show early neuritic branching defects. *Sci. Rep.* **6**, 33377; doi: 10.1038/srep33377 (2016).

## Supplementary Material

Supplementary Information
