# Supplementary material for: Dopaminergic neurons differentiating from LRRK2 G2019S induced pluripotent stem cells show early neuritic branching defects
Source: Sci Rep. 2016 Sep 19;6:33377. doi: 10.1038/srep33377 (PMC5027571; doi:10.1038/srep33377)
Supplement: Supplementary Information [file srep33377-s1.pdf]

Dopaminergic neurons differentiating from *LRRK2* G2019S induced pluripotent stem cells show early neuritic branching defects

Laurence Borgs[1]*, Elise Peyre[1]*, Philippe Alix[1], Kevin Hanon[1], Benjamin Grobarczyk[1], Juliette D. Godin[1], Audrey Purnelle[1], Nathalie Krusy[1], Pierre Maquet[1,2] Philippe Lefebvre[1,3],Vincent Seutin[1], Brigitte Malgrange[1]* and Laurent Nguyen[1]*‡

[1] GIGA-Research, GIGA-Neurosciences, Université de Liège; Belgium. [2] Service de Neurologie, CHU Sart Tilman; Belgium, [3]Service d'othorhinolaryngologie, CHU Sart Tilman; Belgium

* Authors contributed equally to the work

‡ Corresponding author: lnguyen@ulg.ac.be

Supplementary informations and figures

Figure S1

Electrophysiological characterization of cells at different time points of the differentiation protocol

Representation of the different steps of the DA neuron derivation protocol, red arrows points to the two different recording time points: day 10 and day 20 (**A**). Representative current clamp traces of H9 and WT hiPSC differentiating cultures (H9 n=20; hiPSC n=11) (**B**). Representative voltage clamp traces of H9 and WT hiPSCs differentiating cultures. Na^+ currents are blocked by TTX (red trace; wash out: blue trace) and K^+ currents are blocked by TEA (purple trace; wash out: green) (H9 n=20; hiPSC n=11) (**C**). Representative current clamp traces of H9 and WT hiPSC at 20 days of differentiation. Cells where exposed to quinpirole (red trace, wash-out: green) (H9 n=6; hiPSC n=3) (**D**). Representative current clamp traces of H9 and WT hiPSC at 20 days of differentiation. Cells where exposed to ZD 7288 (red trace), green arrows point to the voltage deflexion sag produced by I_h current (H9 n=2; hiPSC n=3) (**E**).

Figure S2

Characterization of the hiPSCs reprogrammed from WT and LRRK2 G2019S patients

Schematic representation of hiPSCs generation (left). Table summarizing the different fibroblast lines reprogrammed (right) (**A**). Representative sequencing of the WT and *LRRK2* G2019S hiPSCs colonies (**B**). qRT-PCR of different pluripotency genes on the different WT and *LRRK2* G2019S fibroblasts and hiPSCs (**C**). Representative immunostaining of WT and G2019S hiPSCs colonies for various pluripotency proteins (**D**). Karyotype of representative WT and G2019S hiPSCs cell lines (**E**).

Figure S3

Microtubule polymerization rate of WT and LRRK2 G2019S NSCs

NSCs nucleofected with pCAGGS-EB3-GFP (left panels) together with a time projection of the same cell to visualize the EB3 comets movements (right panels) for WT and *LRRK2* G2019S cells (**A**). Quantification of the microtubule polymerisation rate for WT and *LRRK2* G2019S cells at different times of culture (**B**).

Figure S1

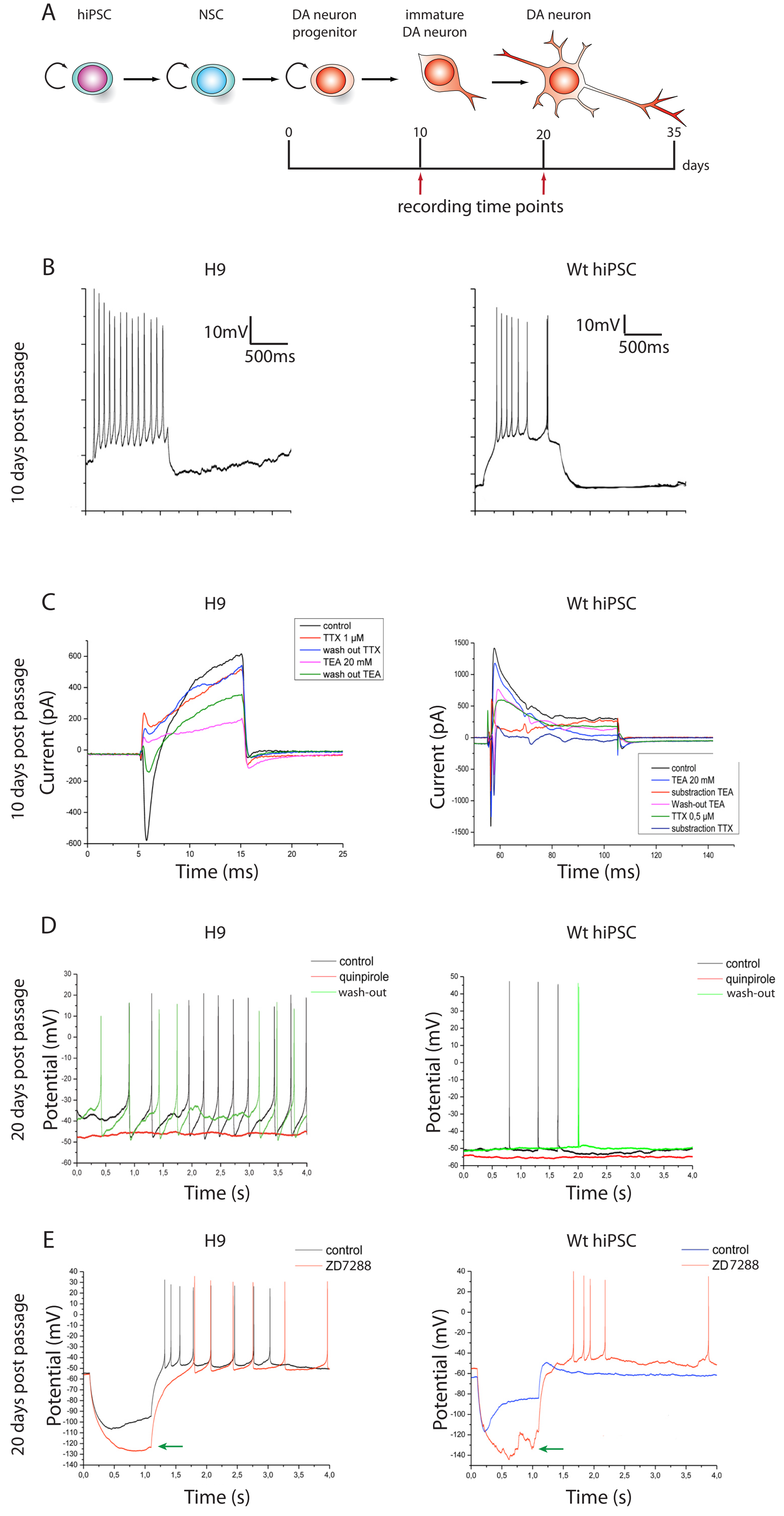
A
hiPSC
NSC
DA neuron progenitor
immature DA neuron
DA neuron
0
10
20
35
days
recording time points
B
H9
Wt hiPSC
10 days post passage
10mV
500ms
C
H9
Wt hiPSC
10 days post passage
Current (pA)
Time (ms)
control
TTX 1 µM
wash out TTX
TEA 20 mM
wash out TEA
TEA 20 mM
substraction TEA
Wash-out TEA
TTX 0,5 µM
substraction TTX
D
H9
Wt hiPSC
20 days post passage
Potential (mV)
Time (s)
control
quinpirole
wash-out
E
H9
Wt hiPSC
20 days post passage
Potential (mV)
Time (s)
control
ZD7288

Figure S2

A

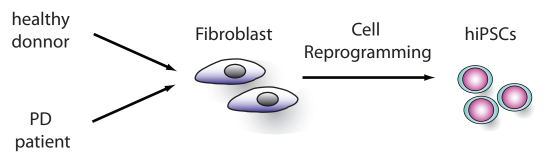

	gender-age	Cell line name
Healthy donor	Women - 37 years old	Wt 01
	Men 50 - years old	Wt 02
	Men - 31 years old	Wt 03
Parkinson's disease patient G2019S mutation of LRRK2	Men 56 - years old	G2019S ND29370
	Men 59 - years old	G2019S 02
	Men 58 - years old	G2019S 03

B

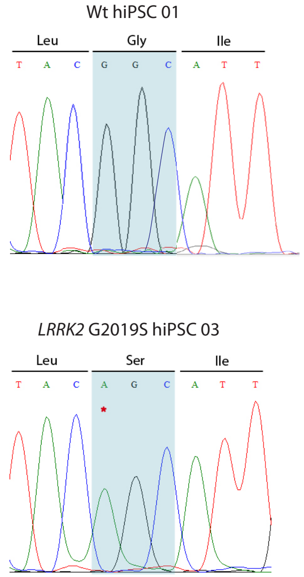

C

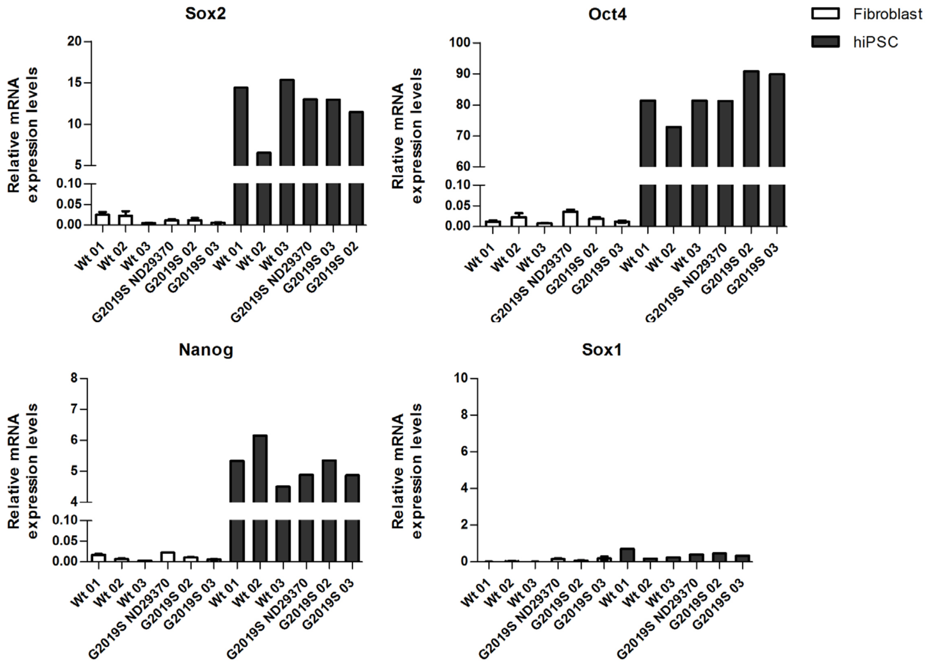

D

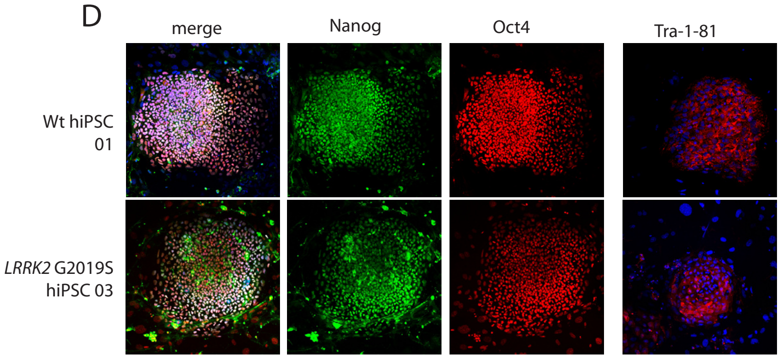

E

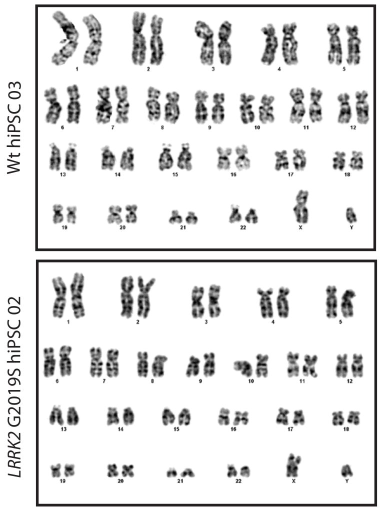

Figure S3

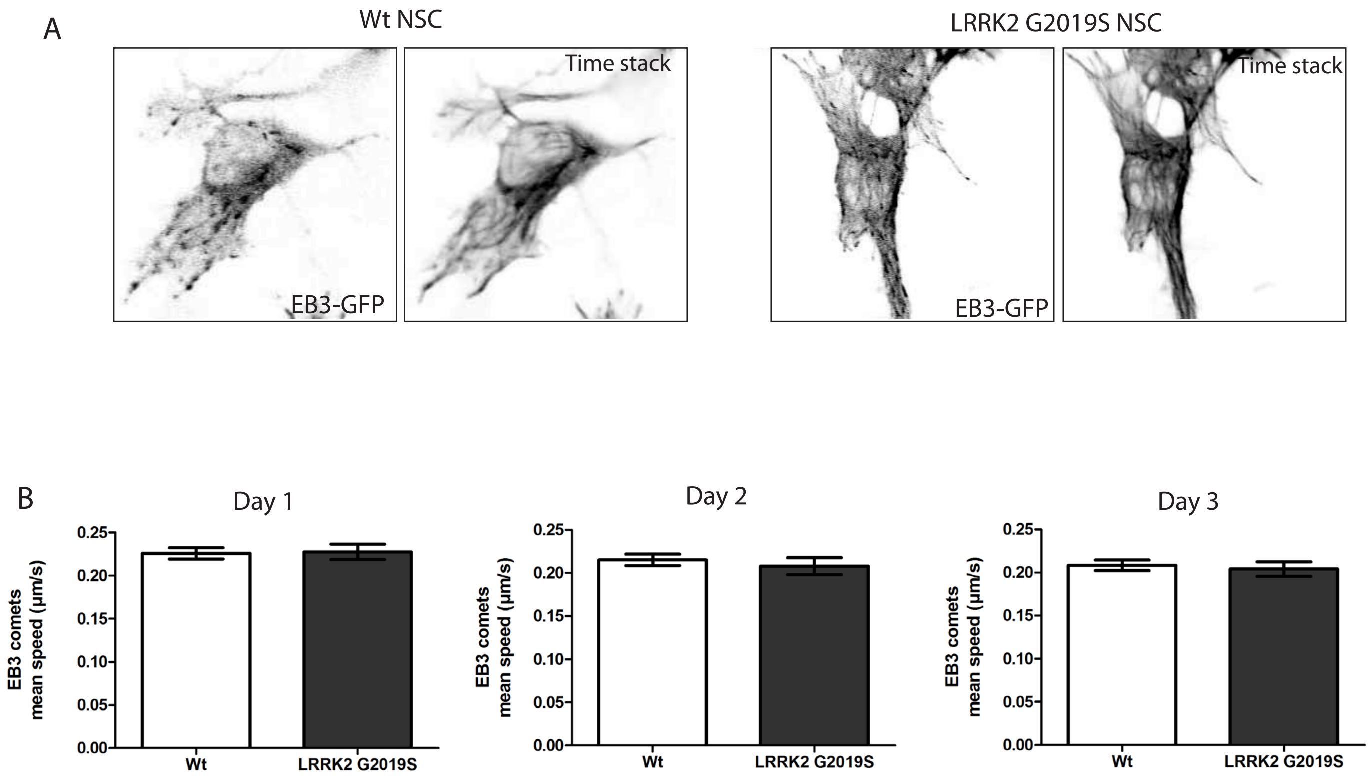
A
Wt NSC
EB3-GFP
Time stack
LRRK2 G2019S NSC
EB3-GFP
Time stack
B
Day 1
Day 2
Day 3
EB3 comets mean speed (µm/s)
0.25
0.20
0.15
0.10
0.05
0.00
Wt
LRRK2 G2019S
